# The impact of diabetes on clinical outcomes in acutely ill patients - a study on patients admitted to the emergency department

**DOI:** 10.1186/s12872-026-06311-9

**Published:** 2026-07-18

**Authors:** Per Wändell, Kean Tang, Emma Kwon, Marcelina Wierzbicka, Karolina Sigurdsson, Caroline Wachtler, Axel C Carlsson, Torgny Wessman, Olle Melander, Ulf Ekelund, Anders Björkelund, Peter M Nilsson, Patrik Rydén, Toralph Ruge

**Affiliations:** 1 Department of Neurobiology, Care Sciences and Society, Division of Family Medicine and Primary Care, Karolinska Institutet, Alfred Nobels allé 23, Huddinge, SE-141 83 Sweden; 2 Center for Primary Health Care Research, Lund University, Malmö, Sweden; 3 Department of Department of Mathematics and Mathematical Statistics, Umeå University, Umeå, Sweden; 4 Department of Emergency and Internal Medicine, Skånes University Hospital, Malmö, Sweden; 5 Department of Clinical Sciences Malmö, Department of Internal Medicine, Lund University, Skåne Malmö, Sweden; 6 Academic Primary Health Care Centre, Region Stockholm, Stockholm, Sweden; 7 Emergency medicine, Department of Clinical Sciences Lund, Department of Emergency Medicine, Lund University, Skåne University Hospital, Lund, Sweden; 8 Department of Clinical Sciences Lund, Lund University, Lund, Sweden; 9 Centre for Environmental and Climate Science, Lund University, Lund, Sweden

**Keywords:** Diabetes, Emergency medicine, Mortality, Multimorbidity, Prediction

## Abstract

**Background:**

Individuals with diabetes have an increased risk of cardiovascular disease, infection, hospitalization, and premature mortality. However, less is known about how diabetes shapes the broader pattern of emergency department (ED) presentations, acute care use, clinical complexity, and short-term mortality in an unselected ED population. We aimed to describe ED presentation patterns and outcomes among individuals with and without diabetes in a large regional cohort.

**Methods:**

We conducted a population-based cohort study including all adult ED visits to nine hospitals in Region Skåne, Sweden, between 2017 and 2018. ED visits for patients with a registered diabetes diagnosis (*n* = 60,654) were compared with those without diabetes (*n* = 502,800). We analysed ED visit frequency, recurrent ED use, arrival by ambulance, triage priority, length of stay, comorbidity burden, presenting complaints, and mortality after ED presentation.

**Results:**

The most common presenting complaints were broadly similar in both groups, with dyspnoea, chest pain, and abdominal pain among the leading causes of ED presentation. However, diabetes visits were characterized by greater acute care complexity. Compared with visits by individuals without diabetes, visits by individuals with diabetes more often involved a previous ED visit within 90 days, higher triage priority, ambulance arrival, longer ED stay, and substantially higher comorbidity burden. Early mortality after ED presentation was also higher among individuals with diabetes and occurred at younger ages, particularly among men. Mortality diagnoses differed between groups, with cardiovascular causes more prominent among individuals with diabetes.

**Conclusions:**

In this large population-based ED cohort, individuals with diabetes presented with broadly similar symptom categories as those without diabetes, but with markedly greater clinical complexity, higher acuity, recurrent acute care use, and earlier mortality. Diabetes in emergency care may therefore identify a patient group with substantial multimorbidity, reduced physiological reserve, and increased vulnerability during acute illness.

**Supplementary Information:**

The online version contains supplementary material available at 10.1186/s12872-026-06311-9.

## Introduction

Diabetes is a chronic condition that has reached global epidemic proportions [1]. Mortality among individuals with diabetes is estimated to be two to four times higher than in those without the disease, and in the United States it ranked as the seventh leading cause of death in 2020 [2]. People living with diabetes are also substantially more likely to require hospital care, with hospitalization rates up to four times higher than in the general population [3]. Approximately 30% of patients discharged with a diabetes diagnosis will experience two or more hospitalizations within a single year [4, 5]. In Sweden, the prevalence of diabetes is roughly 5% [6], and the excess risks of ischemic heart disease [7], heart failure [8, 9], and all-cause mortality are most pronounced below 55 years of age [7, 10, 11].

Emergency departments (EDs) increasingly function as a major point of contact for individuals living with chronic metabolic diseases, and diabetes is one of the most common comorbidities among ED patients. International studies estimate that between 10% and 25% of all ED visits involve people with diabetes [4, 12, 13]. Acute metabolic disturbances such as diabetic ketoacidosis (DKA) and hyperosmolar hyperglycaemic state (HHS) remain important causes of ED utilisation and contribute significantly to morbidity and short-term mortality [14]. Conversely, hypoglycaemia is a frequent cause of emergency visits—particularly among older adults and insulin-treated patients—and is associated with recurrent ED use, hospitalization, and increased mortality risk [5, 15].

Beyond glucose-related emergencies, diabetes is strongly associated with acute infections requiring emergency evaluation, including pneumonia, urinary tract infections, cellulitis, and sepsis [3, 16–18]. Cardiovascular presentations, such as myocardial infarction, heart failure, arrhythmia, and stroke, also occur more frequently among ED patients with diabetes [11, 19–21]. Registry and cohort studies show that individuals with diabetes typically present with higher acuity, greater hemodynamic instability, and more extensive multimorbidity than non-diabetic ED patients. They also have longer ED stays, more frequent use of laboratory and imaging diagnostics, and higher admission rates, even when presenting with similar symptoms [4, 12, 13]. Short-term outcomes—including ICU admission, 30-day readmission, and early mortality—are consistently worse among ED patients with diabetes across a wide range of acute conditions [22–24] .

In the Nordic countries, the burden of diabetes in acute care settings mirrors international patterns; however, these findings should be interpreted within the context of tax-funded, universal healthcare. Swedish registry-based studies demonstrate that individuals with diabetes have substantially higher rates of emergency visits and unplanned hospitalizations compared with those without the condition, particularly due to cardiovascular disease, infections, and acute metabolic complications. Data from the Swedish National Diabetes Register (NDR) further show that acute events, such as myocardial infarction, severe infections, and decompensated heart failure, remain major contributors to morbidity and short-term mortality among adults with diabetes [6, 10, 25]. Danish and Finnish cohort studies report similar findings, with higher ED utilisation, increased illness severity at presentation, longer hospital stays, and elevated 30-day mortality in diabetes patients despite strong primary care access [13]. These observations suggest that, across the Nordic region, diabetes continues to impose a substantial acute care burden, driven by multimorbidity, frailty in older adults, and the high prevalence of cardiovascular and renal complications.

Taken together, these findings suggest that individuals with diabetes represent a large and clinically vulnerable population within emergency care. However, despite extensive evidence regarding increased ED utilization and adverse outcomes, relatively few population-based studies have comprehensively characterized patterns of acute care use, recurrent ED presentations, illness severity, multimorbidity, and mortality across all-cause ED visits among individuals with diabetes.

A clearer understanding of these patterns may improve identification of clinically vulnerable patients and inform emergency care strategies for individuals with diabetes. Therefore, the aim of the present study was to describe ED presentation patterns, acute care utilization, comorbidity burden, and mortality among individuals with and without diabetes in a large population-based emergency care cohort from southern Sweden.

## Methods

### Study design

This study was designed as a population-based cohort analysis using routinely collected data from emergency departments (EDs) in Region Skåne, Sweden. All data were fully anonymized prior to analysis, and the study followed ethical principles and regulatory requirements for secondary use of healthcare data.

### Study population

The dataset (Skåne Emergency Medicine (SEM) database) included all ED visits made by adults aged 18 years and older at nine hospitals in Region Skåne between January 1, 2017, and December 31, 2018. In total, the cohort comprised 563,454 ED visits by 296,991 unique individuals. Of all visits, approximately 49% originated from Malmö and Lund hospitals, 32% from Helsingborg and Kristianstad, and the remaining 19% from the other participating hospitals, as detailed in Table 1.

**Table 1:** Number of ED visits per hospital emergency department in the Skåne cohort among all patients (All), patients with diabetes (DB) and without diabetes (NDB)

Hospital	Total Number of Obs.	DM Obs.	Non-DM
SUS Malmö	153,687	15,810	137,877
SUS Lund	120,905	12,361	108,544
Helsingborg	100,801	10,726	90,075
Central Kristianstad	80,337	8,653	71,684
Ystads	49,537	5,682	43,855
Trelleborg	24,337	3,046	21,291
Hässleholm	18,621	2,199	16,422
Landskrona	10,104	1,457	8,647
Ängelholms	286	32	254
Missing hospital information	4,749	598	4,151
Total	563,364	60,564	502,800

Diabetes status was defined by an indicator variable identifying patients with a previously recorded diagnosis of diabetes prior to the ED visit. Overall, 60,654 (10.8%) of the ED visits were made by patients with a recorded diagnosis of diabetes, corresponding to 36,118 unique individuals.

### Variables of interest

The analyses focus on ED visits. For each visit the following variables were observed for all events and included in the analysis:

- Diabetes status, an indicator variable that is one if the patient had a previously recorded diagnosis of diabetes at the time of arrival.
- Age (18–115 years) and age categories: 18–39, 40–59, 60–79, and ≥ 80 years.
- Sex, an indicator variable that is one if the patient is a women.
- Previous ED visits, an indicator variable that is one if the patient had at least one previous ED visit within 90 days prior to the arrival.
- Arrival by ambulance, an indicator that is one if the patient arrived with ambulance.
- Triage at arrival, a categorical variable following the Rapid Emergency Triage and Treatment System (RETTS). In some analyses this variable was used to construct an indicator variable that was one if the patient received the highest preauthorization, i.e. color code red or orange.
- ED length of stay, measured from arrival to discharge.
- Reason for ED visit, defined as the chief complaint recorded at arrival.
- Comorbidity, defined as the number of diagnosed diseases at the time ofarrival, ignoring diabetes diagnoses, among a set of 258 considered diagnoses.
- Early mortality, an indicator variable that is one if the patient died within 100 days after arrival.

All the above variables were recorded for each visit. It should be stressed that patients can have several visits and that their diabetes status can change over time. In contrast, the main mortality diagnosis, coded according to the ICD-10-CM standard, was linked to the patient’s last ED visit which consequently defined the patient groups.

### Statistical analysis

Through this manuscript patient groups were compared with focus on visits among patients diagnosed with diabetes or not. For continuous variables, including the length of stay at the ED and the considered comorbidity variable, the non-parametric Wilcoxon rank-sum test was used. For analysis focusing on binary outcomes, e.g. percentage of visits with ambulance, the two-sample proportion Z-test was used. For categorical variables, including comparing distribution across age groups, visiting reasons, and mortality diagnosis, the Chi-square test was used. Here, when comparing visiting reasons, and mortality diagnosis, the categorical classes was defined using a two-stage procedure. First the top ten visiting reasons (or mortality diagnosis) was defined for each patient group (patients with or without diabetes), next the union of the top reasons were obtained, and finally a group other was added included all reasons not included in the union set. A p-value less than 0.05 was considered statistically significant for all analyses. P-values below 0.001 are shown as *p* < 0.001. No correction was done for multiple testing. All statistical analyses and visualizations were performed using the Python package SciPy version 1.11.2.

## Results

The distribution of ED visits across the nine hospitals is presented in Table 1.

In total, 563,454 ED visits were made by 296,991 unique patients. 60,654 (10.8%) of the ED visits were made by patients with a recorded diagnosis of diabetes prior to arrival. Older age groups and males were overrepresented among diabetes-related visits compared with non-diabetes visits, see Table 2. Among females, 9.0% of ED visits were made by patients with diabetes, compared with 12.8% among males (ratio = 1.42, *p* < 0.001). The age distributions of diabetes-related and non-diabetes visits differed substantially, with patients with diabetes generally being older (*p* < 0.001). Specifically, 78.9% of diabetes-related visits were made by patients aged 60 years or older, compared with 44.5% of non-diabetes visits (ratio = 1.77, *p* < 0.001).

**Table 2:** Number of ED visits among patients with diabetes (DB) and without diabetes (NDB) stratified by age and sex. For each patient group, defined by age and sex, the number of visits together with the relative distribution between different age group are presented

Sex	Visits	Age groups 18+ 18–39 (%) 40–59 (%) 60–79 (%) 80+ (%)				
All	DB	60,654	5.3	15.8	50.9	28.0
	NDB	502,800	30.1	25.4	29.0	15.5
	Total	563,454				
Female	DB	26,032	6.8	16.0	45.7	31.5
	NDB	262,933	29.9	24.5	28.1	17.5
	Total	288,965				
Male	DB	34,532	4.2	125.7	54.8	25.2
	NDB	239,867	30.3	26.4	30.0	13.3
	Total	274,339				

**Fig. 1:**
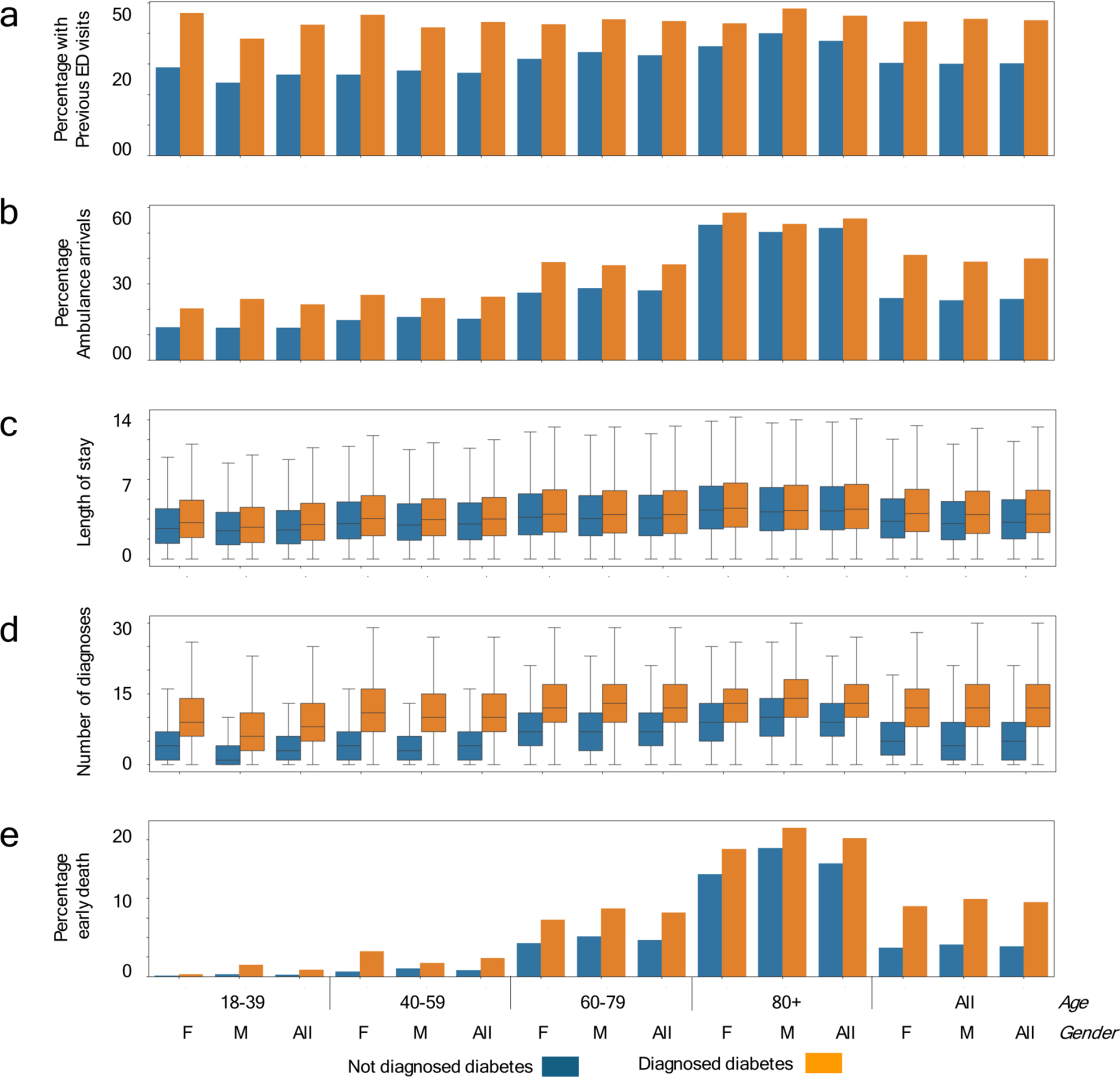
Differences between ED visits by patients with and without diabetes. **a** Percentage of visits preceded by at least one ED visit within the previous 90 days. **b** Percentage of visits arriving by ambulance. **c** Length of stay in the ED (hours). **d** Number of recorded diagnoses at ED arrival. **e** Percentage of visits followed by death within 100 days of the ED arrival. All pairwise comparisons between diabetes and non-diabetes (visits were statistically significant (*p* < 0.001), except for panel (**a**) among females aged 18–39 years, where the corresponding p-value was 0.0393

The frequency of previous ED visits differed between patients with and without diabetes. Overall, 44.3% of visits by patients with diabetes were preceded by at least one ED visit within the previous 90 days, compared with 30.1% of visits by patients without diabetes (ratio = 1.47, *p* < 0.001). This pattern was consistent and statistically significant (*p* < 0.001) across all groups defined by age and sex, see Fig. 1a and Supplementary Table S1.

The reason for visiting ED differed between patients with and without diabetes (*p* < 0.001), see Table 3. Among patients with diabetes, dyspnea, chest pain, and abdominal pain were the three most frequent reasons for visiting the ED. In contrast, among patients without diabetes, abdominal pain was the most common chief complaint, followed by chest pain and dyspnea.

**Table 3:** The ten most common reasons for ED visits among patients with diabetes (DB) and without diabetes (NDB)

Visit Reason DB	Number	Percent	Visit Reason NDB	Number	Percent
Dyspnea	6,691	11.1	Abdominal pain	60,976	12.1
Chest pain	6,300	10.4	Chest pain	44,214	8.8
Abdominal pain	5,071	8.4	Dyspnea	31,160	6.2
Unspecified illness	3,233	5.3	Hand injury	22,880	4.6
Infections	2,666	4.4	Unspecified illness	19,610	3.9
Fever	2,342	3.9	Extremity pain	17,523	3.5
Extremity Pain	2,067	3.4	Head injury	16,833	3.4
Head injury	1,716	2.8	Foot injury	15,056	3.0
Dizziness	1,668	2.8	Dizziness	13,958	2.8
Loss of function	1,624	2.7	Arrhythmia	12,827	2.6
Other reasons	27,276	44,9	Other reasons	247,763	49,3
Total	60,654	100	Total	502,800	100

**Fig. 2:**
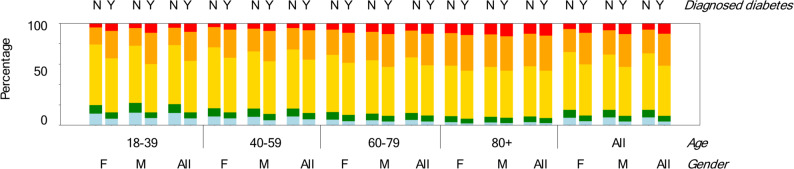
Differences in triage between ED visits by patients with diabetes (Y) and without diabetes (N) stratified by age and sex. The colors denote different priorities: Red (Acute/Life-threating), Orange (Very Soon), Yellow (Urgent), Green (Less acute), and Blue (non-acute)

ED visits by patients with diabetes were more likely to involve arrival by ambulance and to receive high-priority triage classifications than visits by patients without diabetes. Overall, 39.8% of visits by patients with diabetes arrived by ambulance, compared with 24.0% of visits by patients without diabetes (ratio = 1.66, *p* < 0.001). Similarly, 41.8% of visits by patients with diabetes received a high-priority triage classification (red or orange), compared with 29.6% of visits by patients without diabetes (ratio = 1.41, *p* < 0.001). Both patterns were consistent and statistically significant (*p* < 0.001) across all groups defined by age and sex, see Figs. 1b and 2; Supplementary Tables S2 and S3.

Furthermore, patients with diabetes experienced longer stays in the ED than patients without diabetes. The median ED length of stay was 4.48 h for visits by patients with diabetes, compared with 3.68 h for visits by patients without diabetes (ratio = 1.22, *p* < 0.001). This difference was consistently observed across all groups defined by age and sex, see Fig. 1c and Supplementary Table S4.

In total, 25,023 of the 297,191 unique patients who visited the ED died during the study period. Here, the time from arrival at the ED until death ranged from 0 to 1076 days. It should be noted that some patients had multiple ED visits and that their diabetes status could change over time. To account for this, each death was linked to the patient’s last ED visit, and diabetes status was defined according to the status recorded at that visit.

Among the patients who died, 5296 (21.2%) had a recorded diagnosis of diabetes at their last ED visit, whereas 19,727 (78,8%) did not. The distribution of mortality diagnoses differed significantly between these groups (*p* < 0.001). The ten most common mortality diagnoses among deceased patients are presented in Table 4. Among patients with diabetes, acute myocardial infarction was the leading cause of death (5.9%), whereas it ranked second among patients without diabetes (4.8%). In contrast, malignant neoplasm of the bronchus or lung was the most common mortality diagnosis among patients without diabetes (5.5%), while ranking forth among patients with diabetes (3.2%).

**Table 4:** The ten most common causes of death among patients with diabetes (DB) and without diabetes (NDB), where diabetes status was defined according to the patient’s last ED visit prior to death

Death Code DB	Description	Number	Percent	Death Code NDB	Description	Number	Percent
I219	Acute myocardial infarction, unspecified	308	5.9	C349	Malignant neoplasm: Bronchus or lung, unspecified	1,076	5.5
I259	Chronic ischaemic heart disease, unspecified	201	3.8	I219	Acute myocardial infarction, unspecified	941	4.8
I509	Heart failure, unspecified	189	3.6	F03	Unspecified dementia	639	3.2
C349	Malignant neoplasm: Bronchus or lung, unspecified	166	3.2	I509	Heart failure, unspecified	636	3.2
C259	Malignant neoplasm: Pancreas, unspecified	163	3.1	I259	Chronic ischaemic heart disease, unspecified	582	3.0
J449	Chronic obstructive pulmonary disease, unspecified	137	2.6	J449	Chronic obstructive pulmonary disease, unspecified	550	2.8
I482	Chronic atrial fibrillation	124	2.4	C61	Malignant neoplasm of prostate	532	2.7
J189	Pneumonia, unspecified	114	2.2	J189	Pneumonia, unspecified	454	2.3
C61	Malignant neoplasm of prostate	107	2.0	I482	Chronic atrial fibrillation	421	2.1
I64	Acute cerebrovascular disease not specified as hemorrhage or infarction	100	1.9	C189	Malignant neoplasm: Colon, unspecified	401	2.0
Other	Other reasons	3687	69.6	Other	Other reasons	6232	68.4
Total		5296	100	Total		19,727	100

When ED visits rather than unique patients were used as the unit of analysis, early mortality (defined as death within 100 days of the ED visit) was more common after visits by patients with diabetes than after visits by patients without diabetes. Overall, early mortality occurred following 9.5% of diabetes-related visits and 3.9% of non-diabetes visits (ratio = 2.44, *p* < 0.001). This trend was consistent and statistically significant (*p* < 0.001) across all groups defined by age and sex, see Fig. 1e and Supplementary Table S5.

As expected, the risk of early mortality increased with age in both groups. However, the relative difference between patients with and without diabetes was larger among younger patients. For example, among patients aged 40–59 years, the risk of early mortality was 2.4% for patients with diabetes and 0.8% for patients without diabetes (ratio = 3.00, *p* < 0.001). In contrast, among patients aged 80 years or older, the corresponding risks were 17.7% and 14.4%, respectively (ratio = 1.23, *p* < 0.001), see Supplementary Table S5.

Comorbidity, defined as the number of diagnosed diseases at the ED arrival, ignoring diabetes diagnoses, differed significantly between patients with and without diabetes. The median number of diagnosed diseases at the ED arrival was 12.0 for visits for individuals with diabetes, and 5.0 for those without diabetes (ratio = 1.71, *p* < 0.001). This trend was significant in all patient groups considered, see Fig. 2d and Supplementary Table S6.

## Discussion

In this large population-based cohort from nine emergency departments in southern Sweden, we observed substantial differences in acute care utilization, illness severity, multimorbidity, and mortality between individuals with and without diabetes. Although the dominant symptom categories at presentation were broadly similar, individuals with diabetes represented a clinically more vulnerable patient population characterized by recurrent ED use, higher triage acuity, greater ambulance utilization, longer ED stays, higher multimorbidity burden, and earlier mortality.

### Higher ED utilisation among individuals with diabetes

It has previously been shown that the risks of hospitalization, cardiovascular disease, heart failure, and premature mortality are substantially higher among individuals with diabetes, particularly at younger ages [4, 5, 7, 9, 23, 25, 26]. Our findings extend these observations into the emergency department setting. Individuals with diabetes demonstrated substantially greater acute care utilization, including more recurrent ED visits, higher triage acuity, more ambulance arrivals, longer ED stays, and markedly greater multimorbidity burden.

Importantly, although the dominant presenting complaints were broadly similar between groups, individuals with diabetes consistently presented within a more severe and clinically complex context. These findings suggest that diabetes in emergency care is associated not only with chronic metabolic disease, but also with accumulated systemic vulnerability characterized by recurrent acute illness, reduced physiological reserve, and increased short-term mortality.

The earlier mortality observed among individuals with diabetes, particularly among men and at younger ages, further supports the concept of accelerated cardiometabolic vulnerability in this population.

### Diagnostic patterns: similar presentations, greater vulnerability

Dyspnoea, chest pain, abdominal pain, and infections were among the most common reasons for ED presentation in both individuals with and without diabetes. At first glance, the symptom patterns therefore appeared broadly similar between groups. However, the surrounding clinical context differed substantially.

Individuals with diabetes consistently presented with greater multimorbidity, higher triage acuity, more ambulance arrivals, longer ED stays, and markedly higher short-term mortality. Thus, similar presenting symptoms often reflected a fundamentally different degree of clinical complexity and physiological vulnerability in patients with diabetes.

This distinction may be particularly important for infectious presentations. Previous studies have demonstrated increased susceptibility to severe infections, pneumonia, sepsis, and infectious complications among individuals with diabetes [3, 16, 18]. In the present study, infections occurred within a broader pattern of recurrent ED use, greater illness severity, and higher mortality, suggesting that infectious presentations in diabetes frequently develop in the setting of accumulated cardiometabolic disease burden and reduced physiological reserve.

Taken together, the findings suggest that individuals with diabetes frequently present with common ED symptom categories, but within a markedly more vulnerable and high-risk acute care context.

### Comorbidity burden and clinical complexity

One of the most striking findings in the present study was the substantially greater burden of diagnosed disease among individuals with diabetes. The median number of diagnosed diseases at ED presentation was more than twice as high among diabetes visits compared with non-diabetes visits (12 vs. 5 diagnoses), and this pattern was consistent across all age groups and both sexes.

Only 40% of individuals with diabetes had no recorded comorbid disease, compared with more than 70% among individuals without diabetes. Across all comorbidity grades, individuals with diabetes consistently demonstrated a markedly heavier multimorbidity burden. Importantly, these differences were observed even in younger age groups, suggesting that the excess disease burden associated with diabetes develops earlier and accumulates more extensively over time [15, 27–29]. This constellation of chronic disease likely contributes not only to increased ED utilisation but also to longer ED stays, which we observed among individuals with diabetes. Taken together, the findings suggest that diabetes in emergency care rarely occurs as an isolated metabolic disorder. Instead, diabetes appears to identify a patient group characterized by accumulated systemic disease burden, increased clinical complexity, and greater vulnerability during acute illness.

People with diabetes consistently exhibit higher all-cause mortality than the general population, and this excess risk reflects both diabetes-specific pathophysiology and a greater burden of cardiovascular and other chronic conditions [6, 7, 11]. Large nationwide register-based studies from Sweden have demonstrated not only a high and increasing prevalence of diabetes, but also substantial excess mortality among individuals with type 2 diabetes even after adjustment for traditional cardiovascular risk factors and established comorbidities, indicating a residual risk that appears attributable to diabetes per se [6, 7, 11]. In our study, comorbidity was operationalised as a count variable representing the number of pre-existing chronic conditions, capturing the accumulation of cardiovascular disease and heart failure that is commonly observed in people with diabetes and known to adversely affect long-term prognosis [7, 11]. This approach is in line with previous work demonstrating that diabetes substantially accelerates the development and progression of cardiovascular disease and heart failure, which in turn account for a large proportion of the excess mortality among people with diabetes [7, 11]. Notably, diabetes in our emergency department population remained associated with poor clinical outcomes even after adjustment for the comorbidity count, suggesting a residual risk that may be driven by diabetes-specific mechanisms such as long-term hyperglycaemic exposure, micro- and macrovascular damage and altered inflammatory and immune responses [7, 11]. Thus, our findings support the notion that the distinction between “diabetes itself” and “comorbidity” is to some extent artificial: many of the comorbid conditions captured by our comorbidity count are likely downstream consequences of long-standing diabetes, while a diabetes-related risk surplus persists beyond their aggregated burden.

The longer ED stays observed among individuals with diabetes likely further reflect this increased clinical complexity. The mean ED stay was nearly 50 min longer in individuals with diabetes, a finding that may reflect more complicated diagnostic assessments, higher illness severity, greater need for monitoring and investigations, or more challenging disposition decisions—patterns well documented in multimorbid populations [3, 30].

### Severity of presentation and ambulance transport

Transport patterns also differed substantially between groups. Individuals with diabetes were significantly more likely to arrive by ambulance and more frequently received high-priority triage classifications, suggesting greater illness severity at presentation and increased acute physiological instability. Previous studies have similarly shown that individuals with diabetes who require acute care often present with more pronounced physiological disturbances, including hemodynamic instability, electrolyte abnormalities, and signs of infection [16, 17, 31, 32].

### Mortality patterns and age differences

Early mortality after ED presentation was substantially higher among individuals with diabetes and occurred at younger ages, particularly among men. These findings are consistent with previous epidemiological studies demonstrating that diabetes is associated with accelerated cardiovascular disease development, earlier multimorbidity accumulation, and excess mortality at younger ages [10, 33, 34]. While the leading causes of death were broadly similar, notable differences emerged. Pancreatic cancer ranked among the top five mortality diagnoses in individuals with diabetes but did not appear among the ten most common causes in the non-diabetic population. Conversely, unspecified dementia ranked third among individuals without diabetes but only ninth among those with diabetes. The elevated ranking of pancreatic cancer in the diabetes group is highly consistent with international epidemiology. Meta-analyses and large cohort studies from Europe, the United States, and Asia show that diabetes is associated with a 1.5–2.5-fold increased risk of pancreatic cancer and poorer survival after diagnosis [35–37]. Individuals with diabetes are disproportionately represented among those who die from pancreatic cancer, reinforcing that our findings reflect a well-established global pattern. In our cohort, pancreatic cancer ranked as the fifth most common cause of death among individuals with diabetes, whereas it did not appear among the ten leading causes of death in participants without diabetes. This pattern is likely to reflect the complex bidirectional relationship between diabetes and pancreatic cancer, including both long-standing diabetes as a risk factor for pancreatic cancer and pancreatic cancer– or treatment-induced diabetes following pancreatic surgery or other pancreatic damage. Despite its high case fatality, pancreatic cancer still accounted for only a relatively small proportion of all deaths in the diabetic population.

In contrast, dementia ranking higher among individuals without diabetes is also in accordance with international evidence. Dementia mortality is strongly age-dependent, and individuals without diabetes more frequently survive to very old age. Studies show that the excess mortality associated with diabetes declines substantially after age 75 [38]. Thus, fewer individuals with diabetes survive into the age window where dementia becomes a predominant cause of death. This pattern reflects competing risks, particularly earlier cardiovascular and cancer-related mortality in diabetes.

Chronic atrial fibrillation was also more prominent as a cause of death in the diabetes group, consistent with international studies showing that diabetes increases the risk of atrial fibrillation, structural heart disease, and thromboembolic complications [8, 9, 39, 40].

A striking finding was the higher rate of mortality among men with diabetes aged 60–79 years, whereas individuals without diabetes showed the highest mortality among women aged ≥ 80 years. These patterns mirror earlier studies demonstrating that the excess mortality risk associated with diabetes is greatest below age 75, after which mortality curves converge [40]. Thus, the elevated mortality observed among men with diabetes aged 60–75 years likely reflects both a higher biological vulnerability and a greater absolute burden of cardiometabolic disease in this group.

Taken together, the findings suggest that diabetes in emergency care is associated not only with increased mortality, but also with earlier and more clinically complex trajectories of acute deterioration.

### Interpretation and clinical implications

Recent nationwide Swedish data suggest that much of the excess cardiovascular and mortality risk associated with diabetes may be driven by accumulated disease burden and diabetes duration rather than diabetes type alone. Our findings extend this concept to the emergency care setting, where individuals with diabetes presented with markedly greater multimorbidity, higher acuity, and earlier mortality despite broadly similar presenting complaints. Additionally, our data indicate that diabetes is associated not only with chronic cardiometabolic disease, but also with markedly different trajectories during acute illness.

Our findings highlight several concrete opportunities to improve the care of acutely ill patients with diabetes in routine practice. In our study, diabetes emerged as a clear marker of increased risk for adverse outcomes, which supports more systematic risk stratification already at triage, where known diabetes, in combination with age, vital signs and comorbidities, could function as a “red flag” prompting prioritized assessment, closer monitoring and a lower threshold for admission. This is consistent with the current use of structured early warning scores, such as NEWS, to detect deterioration in acutely ill patients, and may be complemented by more consistent documentation of diabetes status, glycaemic control and cardiometabolic risk factors in the emergency record. In light of our results, “tailored strategies” for patients with diabetes also become more tangible: standardized triage and monitoring protocols in which diabetes is included as a risk variable; structured medication review during the acute phase (for example corticosteroids, SGLT2 inhibitors and insulin adjustments); and planned follow-up in primary care for clearly defined high-risk patients after discharge. These measures align with international recommendations on multifactorial risk reduction in diabetes, where optimized control of glucose, blood pressure and lipids, together with organized secondary prevention and systematic follow-up, are emphasized. Taken together, our results support viewing diabetes as a marker of vulnerability in acutely ill patients, for whom systematic routines for risk identification, optimization of cardiometabolic treatment and organized communication and follow-up with primary care after an emergency visit may help reduce the broad excess mortality we observe, rather than focusing exclusively on specific diagnoses.

### Strengths and limitations

Strengths include the large, population-based cohort, high completeness, and standardized ED data. Limitations include lack of information on diabetes duration, glycemic control, disease severity, and social determinants of health. Another limitation is that the study combined data from nine emergency departments that differed in size and catchment populations, ranging from large university hospitals to smaller regional hospitals. Although all participating hospitals operated within the same publicly funded healthcare system and used the same regional triage framework (RETTS), differences in referral patterns, case-mix, and patient complexity may have influenced some outcomes. Hospital-specific analyses were beyond the scope of the present study, and residual confounding related to hospital-level characteristics cannot be excluded.

## Conclusion

Individuals with diabetes presenting to the emergency department exhibited substantially greater multimorbidity, higher acute care utilization, increased illness severity, and higher short-term mortality compared with individuals without diabetes. Although presenting complaints were broadly similar between groups, patients with diabetes consistently presented within a more clinically vulnerable and complex acute care context. Mortality risk was also greater at younger ages among individuals with diabetes. Together, these findings suggest that diabetes in emergency care may serve as a marker of accumulated systemic vulnerability and high-risk acute illness trajectories.

## Supplementary Information

Supplementary Material 1.

## Data Availability

The technical appendix, statistical code, and datasets generated and/or analysed during the current study are available from the corresponding author upon reasonable request.
