# Supplementary material for: The impact of diabetes on clinical outcomes in acutely ill patients - a study on patients admitted to the emergency department
Source: BMC Cardiovasc Disord. 2026 Jul 18;26:609. doi: 10.1186/s12872-026-06311-9 (PMC13379973; doi:10.1186/s12872-026-06311-9)
Supplement: Supplementary file 1 — Supplementary Material 1. [file 12872_2026_6311_MOESM1_ESM.docx]

## Supplementary Tables

**Table S1:** Percentage of ED visits in which the patient had at least one previous ED visit during the preceding 90 days among patients with diabetes (DB) and without diagnosed diabetes (NDB), stratified by age and sex. All the DB-NDB differences in the par wise comparisons were highly significant (p<0.001).

| Sex | Patients | Age groups | | | | |
| --- | --- | --- | --- | --- | --- | --- |
|  |  | 18+ (%) | 18-39 (%) | 40-59 (%) | 60-79 (%) | 80+ (%) |
| All | DB  NDB | 44.3  30.1 | 42.8  26.4 | 43.7  27.1 | 43.9  32.7 | 45.7  37.5 |
| Female | DB  NDB | 43.8  30.2 | 46.6  28.8 | 46.0  26.4 | 43.0  31.6 | 43.2  35.7 |
| Male | DB  NDB | 44.7  30.0 | 38.2  23.8 | 41.8  27.8 | 44.5  33.9 | 48.0  40.0 |

**Table S2:** Percentage of ED visits in which the patient arrived by ambulance among patients with diabetes (DB) and without diagnosed diabetes (NDB) stratified by age and sex. All the DB-NDB differences in the par wise comparisons were highly significant (p<0.001).

| Sex | Patients | Age groups | | | | |
| --- | --- | --- | --- | --- | --- | --- |
|  |  | 18+ (%) | 18-39 (%) | 40-59 (%) | 60-79 (%) | 80+ (%) |
| All | DB  NDB | 39.8  24.0 | 21.9  12.7 | 24.9  16.3 | 37.7  27.4 | 55.6  51.9 |
| Female | DB  NDB | 41.3  24.4 | 20.2  12.8 | 25.6  15.6 | 38.4  26.4 | 57.9  53.1 |
| Male | DB  NDB | 38.7  23.5 | 24.0  12.6 | 24.3  17 | 37.2  28.3 | 53.5  50.2 |

**Table S3:** Triage at the ED for visits by patients with diabetes (DB) and without diabetes (NDB). For each patient group, defined by age and sex, the percentage of visits with the highest prioritization (either red or orange) are presented together with the associated p-value. All the DB-NDB differences in the par wise comparisons were highly significant (p<0.001).

| Sex | Patients | Age groups | | | | |
| --- | --- | --- | --- | --- | --- | --- |
|  |  | 18+ (%) | 18-39 (%) | 40-59 (%) | 60-79 (%) | 80+ (%) |
| All | DB  NDB | 41.8  29.6 | 37.1  21.6 | 35.9  25.8 | 41.4  33.7 | 46.7  42.2 |
| Female | DB  NDB | 40.3  28.4 | 34.7  21.1 | 33.9  23.9 | 38.9  31.1 | 46.6  41.6 |
| Male | DB  NDB | 42.9  30.9 | 40.0  22.2 | 37.5  27.9 | 42.9  36.3 | 46.8  43.1 |

**Table S4:** Median length of stay at the ED (in hours) for visits by patients with diabetes (DB) and without diabetes (NDB). For each patient group, defined by age and sex, the median and interquartile range are presented. All the DB-NDB differences in the par wise comparisons were highly significant (p<0.001).

| Sex | Patients | Age groups | | | | |
| --- | --- | --- | --- | --- | --- | --- |
|  |  | 18+ | 18-39 | 40-59 | 60-79 | 80+ |
| All | DB  NDB | 4.48 (4.26)  3.68 (3.92) | 3.45 (3.73)  2.93 (3.40) | 4.02 (3.87)  3.50 (3.68) | 4.48 (4.27)  4.12 (4.08) | 4.98 (4.42)  4.83 (4.35) |
| Female | DB  NDB | 4.55 (4.28)  3.78 (3.98) | 3.66 (3.78)  3.05 (3.47) | 4.07 (4.03)  3.57 (3.73) | 4.52 (4.23)  4.18 (4.13) | 5.08 (4.45)  4.90 (4.35) |
| Male | DB  NDB | 4.43 (4.23)  3.57 (3.85) | 3.20 (3.52)  2.82 (3.30) | 3.97 (3.75)  3.43 (3.65) | 4.47 (4.28)  4.05 (4.05) | 4.88 (4.45)  4.75 (4.33) |

**Table S5:** Percentage of ED visits in which the patient died within 100 days after arrival among patients with diabetes (DB) and without diagnosed diabetes (NDB) stratified by age and sex. All the DB-NDB differences in the par wise comparisons were highly significant (p<0.001) except for females age 18-39 where the observed p-value was 0.0393.

| Sex | Patients | Age groups | | | | |
| --- | --- | --- | --- | --- | --- | --- |
|  |  | 18+ (%) | 18-39 (%) | 40-59 (%) | 60-79 (%) | 80+ (%) |
| All | DB  NDB | 9.5  3.9 | 0.8  0.2 | 2.4  0.8 | 8.2  4.7 | 17.7  14.4 |
| Female | DB  NDB | 9.0  3.7 | 0.3  0.1 | 3.2  0.6 | 7.3  4.2 | 16.3  13.1 |
| Male | DB  NDB | 9.9  4.1 | 1.5  0.2 | 1.7  1.0 | 8.7  5.1 | 19.0  16.4 |

**Table S6:** Median number of diagnosed diseases (diabetes excluded) for ED visits among patients with diabetes (DB) and without diabetes (NDB). For each patient group, defined by age and sex, the median and interquartile range are presented. All the DB-NDB differences in the par wise comparisons were highly significant (p<0.001).

| Sex | Patients | Age groups | | | | |
| --- | --- | --- | --- | --- | --- | --- |
|  |  | 18+ (%) | 18-39 (%) | 40-59 (%) | 60-79 (%) | 80+ (%) |
| All | DB  NDB | 12.0 (9.0)  5.0 (8.0) | 8.0 (8.0)  3.0 (5.0) | 10.0 (8.0)  4.0 (6.0) | 12.0 (8.0)  7.0 (7.0) | 13.0 (7.0)  9.0 (7.0) |
| Female | DB  NDB | 12.0 (8.0)  5.0 (7.0) | 9.0 (8.0)  4.0 (6.0) | 11.0 (9.0)  5.0 (6.0) | 12.0 (8.0)  7.0 (7.0) | 13.0 (7.0)  9.0 (8.0) |
| Male | DB  NDB | 12.0 (9.0)  4.0 (8.0) | 6.0 (8.0)  1.0 (4.0) | 10.0 (8.0)  3.0 (5.0) | 13.0 (8.0)  8.0 (8.0) | 14.0 (8.0)  10.0 (8.0) |
